# Alcohol Consumption Is a Coping Mechanism for Male Patients with Severe Anxiety Disorders Treated with Antidepressants Monotherapy

**DOI:** 10.3390/jcm13092723

**Published:** 2024-05-06

**Authors:** Mădălina Iuliana Mușat, Felicia Militaru, Ion Udriștoiu, Smaranda Ioana Mitran, Bogdan Cătălin

**Affiliations:** 1 U.M.F. Doctoral School Craiova, University of Medicine and Pharmacy of Craiova, 200349 Craiova, Romania; madalina.musat3@gmail.com; 2 Experimental Research Centre for Normal and Pathological Aging, University of Medicine and Pharmacy of Craiova, 200349 Craiova, Romania; bogdan.catalin@umfcv.ro; 3 Department of Psychiatry, University of Medicine and Pharmacy of Craiova, 200349 Craiova, Romania; feliciobanu@yahoo.com; 4 Department of Physiology, University of Medicine and Pharmacy of Craiova, 200349 Craiova, Romania

**Keywords:** anxiety, antidepressants, liver enzymes, blood glucose, alcohol

## Abstract

**Background:** Anxiety disorders are prevalent mental health conditions often accompanied by various comorbidities. The association between anxiety and liver disease, as well as fluctuations in blood sugar levels, highlights the importance of carefully evaluating patients with anxiety undergoing antidepressant therapy. The aim of this study was to conduct a comparative assessment of liver function and blood glucose levels in patients diagnosed with anxiety disorders while considering potential gender-specific differences. **Methods:** An analysis was conducted over a 24-month period. This study included 88 patients diagnosed with anxiety disorders, with symptoms severe enough to require hospitalization, aged 18 or older, undergoing antidepressant monotherapy, without any additional pathologies. Liver enzymes (AST, ALT, GGT), AST/ALT ratio, and blood glucose levels were measured and compared. **Results:** While no significant differences were found between antidepressant classes, increased GGT levels were observed in men older than 40 years compared to women of the same age, suggesting that alcohol consumption may be a coping mechanism for anxiety. This gender difference was not observed among young patients. **Conclusions:** Early detection of alcohol consumption is essential in patients with anxiety disorders in order to prevent alcohol-related liver damage and to adjust the management of both conditions accordingly.

## 1. Introduction

Anxiety disorders are mental health conditions that often coexist with various medical comorbidities [1]. The presence of anxiety disorders can be associated with several pathologies such as hypertension [2], elevated blood glucose levels in diabetic patients [3], irritable bowel syndrome [4], and thyroid dysfunction [5]. Independently, the presence of non-alcoholic fatty liver disease (NAFLD) increases the risk of developing anxiety [6,7], with the histological severity of NAFLD also being correlated with the presence of anxiety [8]. This bidirectional association underscores the need for integrated approaches [9] to assess and manage both hepatic status and mental conditions in affected individuals.

Managing anxiety may become a true challenge, particularly given the high prevalence of comorbid depression among individuals with anxiety disorders [10]. Patients diagnosed with anxiety disorders frequently report insomnia [11,12], impaired social adaptation [13,14], and diminished quality of life [15,16], highlighting the complex nature of anxiety disorders and the importance of a comprehensive therapeutic approach. Moreover, the presence of depression in individuals with anxiety disorders is associated with a higher risk of suicidality and poorer overall prognosis [17]. However, the treatment of anxiety may potentially lead to drug-induced liver injury (DILI) [18].

With female patients having a notable correlation between anxiety and a severe stage of steatosis [19], with the known correlation between anxiety and NAFLD or alcoholic liver disease in men [20], and with the incidence of DILI independently associated with anxiety [21], the evaluation of liver function of those suffering from anxiety appears to be a mandatory step in the treatment strategy. This is particularly true as most pharmacological treatments of anxiety involve antidepressants (ADs) [22]. With selective serotonin reuptake inhibitor(s) (SSRIs) and selective serotonin-norepinephrine reuptake inhibitor(s) (SNRIs) being the first-line psychopharmacologic treatment in anxiety disorders [23], special attention should be directed towards their administration, although they have been associated with a lower risk of hepatotoxicity compared to other ADs [18]. Additionally, alcohol use and anxiety disorders often coexist, and can further complicate the management of both conditions [24]. Alcohol consumption in patients with anxiety is often associated with adverse physical health outcomes and increased mortality risk [25]. Individuals with anxiety disorders may turn to alcohol to relieve feelings of anxiety or distress, leading to the development of alcohol use disorders (AUDs) [26]. Conversely, chronic alcohol consumption can induce or exacerbate symptoms of anxiety through dysregulation of stress response systems and alterations in neurotransmitter function [27].

The management of anxiety is further complicated due to the manner in which alcohol consumption can alter blood glucose levels. Alcohol consumption can lead to acute hypoglycemia by inhibiting gluconeogenesis [28], and chronic alcohol abuse can exacerbate insulin resistance, leading to persistent hyperglycemia and an increased risk of developing diabetes [29]. Although it may seem minor, fluctuations in blood sugar levels can worsen anxiety symptoms and contribute to mood instability [30], further complicating the management of anxiety disorders. Moreover, individuals with anxiety disorders may be more susceptible to the mood-altering effects of alcohol, leading to negative emotionality and impulsivity [31]. This emotional response, combined with the disruptive effects of alcohol on blood sugar control, can create a cyclical pattern of alcohol use and worsening anxiety symptoms.

Gender disparities significantly influence both anxiety disorders and alcohol consumption. While anxiety disorders are more prevalent in women compared to men [32,33], men tend to have higher rates of alcohol intake and AUDs [34]. Women often report different symptom profiles and coping mechanisms in response to stress compared to men [35], experiencing more internalized symptoms, such as rumination and anxiety, whereas men may cope with stress through behaviors such as substance use or aggression [36]. Biological, psychological, and sociocultural factors contribute to these gender differences, including hormonal fluctuations and gender-specific socialization experiences [34]. However, it is essential to recognize that women are not immune to alcohol-related problems, and the gender difference in alcohol consumption is narrowing, particularly among the younger population [37,38]. As most clinicians understand that self-reported alcohol consumption is highly variable and cannot be taken into account in its entirety, liver tests are necessary to determine if patients minimize their intake with most of the studies looking into self-reporting advising for additional objective testing or improving the methodology [39,40,41,42,43].

While previous studies have explored the impact of ADs on liver function or blood glucose levels [44,45,46,47], and some have separately examined the effects of chronic or abusive alcohol consumption on these parameters [48,49,50,51,52,53], there is a significant gap in understanding the combined effects of ADs monotherapy and moderate alcohol consumption on hepatic and glucose metabolism in patients with severe anxiety disorders. Addressing this gap is vital for optimizing treatment strategies and enhancing the overall care and management of hospitalized patients with anxiety disorders who are receiving ADs monotherapy and consuming alcohol in moderate amounts.

The combination of anxiety and physiological factors requires a careful analysis in order to facilitate targeted interventions and complex patient care. The aim of this study was to conduct a comparative assessment of hepatic status and blood glucose levels among patients diagnosed with anxiety disorders, severe enough to require hospitalization, and undergoing monotherapy treatment with various ADs, taking also into account potential gender-based variations, in order to bypass the subjectivity inherent in patients’ self-assessment of their alcohol consumption and the challenges clinicians encounter when treating patients with anxiety in a population who report moderate alcohol intake. By focusing on this subgroup, we intended to investigate alcohol consumption as a coping mechanism for anxiety, rather than addressing the broader population of patients with anxiety disorders. While the subset of patients in our study may be considered undertreated, it is precisely this population that may be more inclined to use alcohol as a coping mechanism, thereby highlighting the importance of early detection and appropriate management of anxiety disorders. This study was conducted in order to generate new hypotheses that can guide future research efforts aimed at optimizing treatment strategies and improving the overall care and management of patients with severe anxiety disorders who are receiving AD monotherapy and consuming alcohol in moderate amounts, including gender-specific impact, the effect of age on liver function, and potential interactions between alcohol consumption, antidepressant treatment, and glucose metabolism, considering factors such as insulin resistance, hormonal dysregulation, and dietary habits among this particular category of patients.

## 2. Materials and Methods

### 2.1. Sample

Research was undertaken using medical records over a period of 24 months (1 January 2022–1 January 2024) in Psychiatry Clinic I—Neuropsychiatry Hospital of Craiova. This study was conducted according to the guidelines of the local Ethics Committee of the University of Medicine and Pharmacy of Craiova (no 67/20 April 2022) and the Ethical Council of Neuropsychiatry Hospital of Craiova (no 2/2 May 2022), under the Romanian and European laws, in accordance with Helsinki ethical guidelines. Specific ethical considerations addressed in the study included ensuring patient confidentiality by anonymizing all data and obtaining informed consent from all participants. This study did not include patients whose mental impairment and discernment could have interfered with their ability to understand their rights and provide informed consent. Data handling procedures were also conducted in compliance with data protection regulations to safeguard the privacy and security of patient information.

### 2.2. Inclusion and Exclusion Criteria

This study included individuals aged 18 years or older, diagnosed with anxiety disorders, according to the International Classification of Diseases, Tenth Revision (ICD 10) criteria [54], regardless of gender. All participants were treated with antidepressants in monotherapy and had no additional pathologies. Patients with coexisting medical conditions, alcohol abuse, and alcoholism were excluded from this study. To confirm their exclusion, the patients underwent thorough medical examinations and a detailed anamnesis regarding alcohol consumption. The patients included in our study were patients who described their alcohol consumption as occasional, in moderate amounts, maximum 2 drinks per day (0.22 to 1.00 fl oz alcohol per day), not every day [55]. Individuals with coexisting medical conditions may present confounding variables that could influence the outcomes of the study. Also, individuals with liver disease, or other chronic conditions may metabolize alcohol differently or experience different reactions to alcohol consumption [56]. Excluding individuals with coexisting medical conditions ensures that the study’s results are more accurate and directly attributable to the effects of moderate alcohol consumption, rather than the influence of other medical conditions. Excluding individuals with alcoholism controls the effects of alcohol dependence on this study’s outcomes. It ensures that the results are not heavily influenced by the specific characteristics and responses of individuals with alcohol dependence. We aimed to control for these confounding variables and ensure that this study’s results are more accurate, reliable, and generalizable to the broader population.

**Figure 1:**
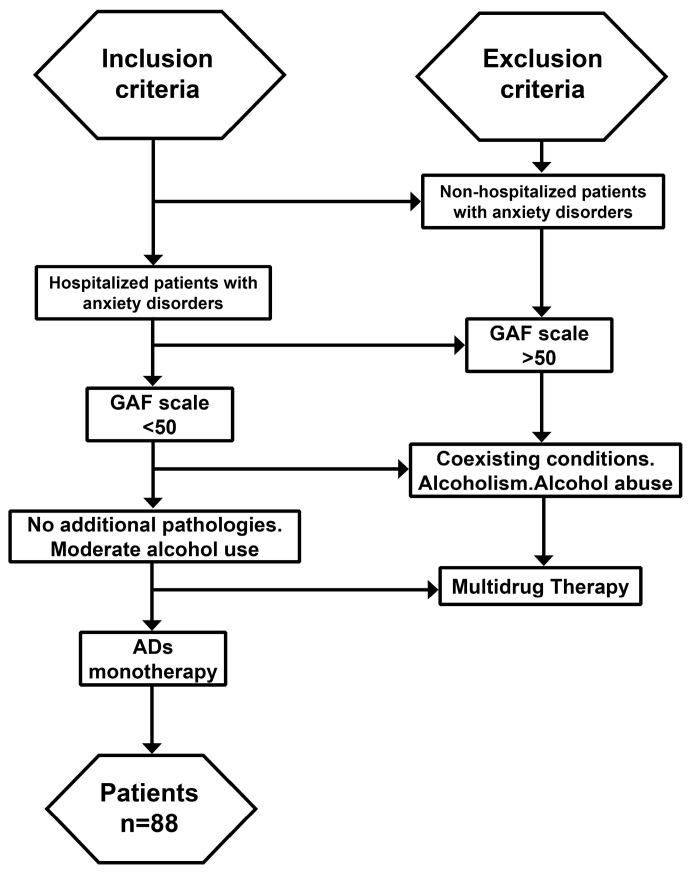
Flow chart of anxiety-diagnosed patients selected for this study. We included patients with anxiety disorders severe enough to require hospitalization, with a GAF scale < 50 at the time of admission. Patients with concomitant medical conditions identified through medical investigations and history, as well as those with alcoholism or alcohol abuse, were excluded. The patients included in the study had self-reported moderate alcohol consumption and were treated with antidepressants in monotherapy at the time of sample collection. A total of 88 patients met the inclusion criteria.

Functional impairment among the subjects was assessed using The Global Assessment of Functioning (GAF) scale [57]. All patients included in the study had a GAF scale below 50 and a symptomatology severe enough to require hospitalization (Figure 1). The typical hospitalization period of the patients with anxiety was around one week. The total number of participants included in this study after applying the inclusion and exclusion criteria was 88.

### 2.3. Antidepressant Treatment

Patients received monotherapy treatment with ADs for at least 3 months in usual daily dosages. The classes of antidepressants administered were as follows: selective serotonin reuptake inhibitor(s) (SSRIs) (escitalopram 10 mg, paroxetine 20 mg, sertraline 50 mg), selective serotonin-norepinephrine reuptake inhibitor(s) (SNRIs) (duloxetine 60 mg, venlafaxine 75 mg), atypical tricyclic antidepressants (TCAs) (tianeptine 37.5 mg), and serotonin antagonist and reuptake inhibitor (SARI) (trazodone 150 mg).

### 2.4. Clinical and Biochemical Evaluations

We collected relevant medical and psychiatric history, medication received, results of blood glucose level (Glucose kit, BioSystems, 21503) and liver enzyme values: gamma-glutamyl transferase (GGT) (gamma-GT kit, BioSystems, 21520), aspartate aminotransferase (AST) (Aspartate Aminotransferase (AST/GOT) kit, BioSystems, 21531), alanine aminotransferase (ALT) (Alanine Aminotransferase (ALT/GPT) kit, BioSystems, 21533) and AST/ALT ratio value. The samples were processed with the BioSystems BA400 Smart Efficiency Biochemistry Analyzer (BioSystems S.A., Costa Brava 30 08030, Barcelona, Spain).

Taking in consideration the recommendation of self-reporting alcohol consumption studies [39,40,41,42,43], we additionally measured the levels of GGT, AST, and ALT and calculated the AST/ALT ratio. This allowed us to have an objective measure of alcohol intake irrespective of the patient’s self-reporting [58,59,60].

### 2.5. Statistical Analysis

Statistical analysis was performed using GraphPad 10.1 and Microsoft Excel 2016 and figures were generated with Adobe InDesign 2024. Normality testing was conducted through the D’Agostino and Pearson test, employing an alpha value of 0.05. Mean differences among the groups were examined using Kruskal–Wallis with multiple comparisons and a two-stage linear set-up, along with the Kolmogorov–Smirnov test. The difference was considered if the value of *p* (Kolmogorov–Smirnov test)/*q* (Kruskal–Wallis test) was under 0.05. All figures show the mean value and standard deviation (SD). Comparisons use ** < 0.01.

## 3. Results

### 3.1. No Differences in Liver Enzymes and Blood Glucose Levels Were Observed in Patients with Anxiety, Regardless of Treatment

A total of 88 patients (36 male and 53 female) met the inclusion criteria. The study group had an average age of 50.00 ± 11.15 years. Of the total number of patients, 29 were treated with SSRI, 26 with SNRI, 17 with TCA, and 16 with SARI (Table 1).

The average values of the measured biological parameters were within the acceptable range considered normal. Gaussian distribution was not observed in any of the tested parameters (Table 2).

**Figure 2:**
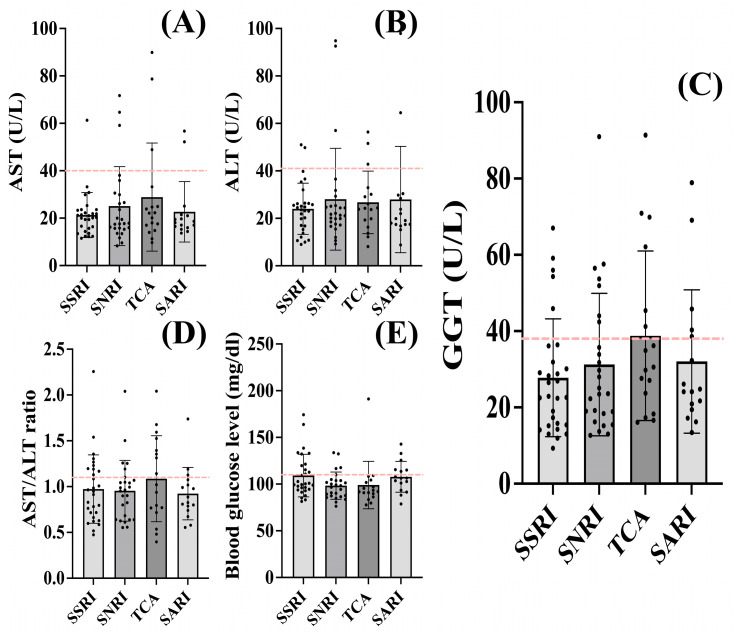
Liver enzyme levels according to treatment received. No differences in (**A**) AST, (**B**) ALT, (**C**) GGT, (**E**) blood glucose levels, and (**D**) AST/ALT ratio regarding the treatment were observed. A trend of elevated AST (28.89 ± 22.82 U/L), GGT (38.77 ± 22.23 U/L) levels, and AST/ALT ratio (1.086 ± 0.4696) were observed in patients undergoing atypical TCA treatment, although they were statistically not significant when compared to the other ADs (*q* > 0.05). Regardless of the treatment administered, the patients showed similar blood glucose levels (*q* > 0.05). The dash line indicates the upper limit of normality for the biological parameters. The graph shows mean values ± SD.

Upon assessing the variations in liver enzymes and blood glucose levels among the studied classes of ADs, no differences were found (Figure 2).

We observed a tendency of elevated AST (28.89 ± 22.82 U/L), GGT (38.77 ± 22.23 U/L) levels, and AST/ALT ratio (1.086 ± 0.4696) in patients who received atypical TCA treatment, but statistically insignificant when compared to the other ADs (*q* > 0.05) (Figure 2A,C,D). The most important difference in GGT enzyme values was noticed in patients treated with atypical TCA (38.77 ± 22.23 U/L), compared to SSRIs (27.76 ± 15.43 U/L), but statistically insignificant (*q* = 0.2998) (Figure 2C). ALT assessment in atypical TCA-treated patients (26.74 ± 13.19 U/L) showed similar values with SSRIs (24.00 ± 10.78 U/L), SNRIs (28.05 ± 21.44 U/L) and SARIs (27.93 ± 22.39 U/L) (*q* > 0.05) (Figure 2B). The same phenomenon was also observed regarding the blood glucose level in atypical TCA-treated patients (98.98 ± 25.39 mg/dL) compared to SSRIs (109.2 ± 22.81 mg/dL), SNRIs (98.33 ± 14.60 mg/dL) and SARIs (107.8 ± 16.55 mg/dL) (*q* > 0.05) (Figure 2E).

### 3.2. Increased GGT Values Were Observed in Men Compared to Women Regardless of Treatment

**Figure 3:**
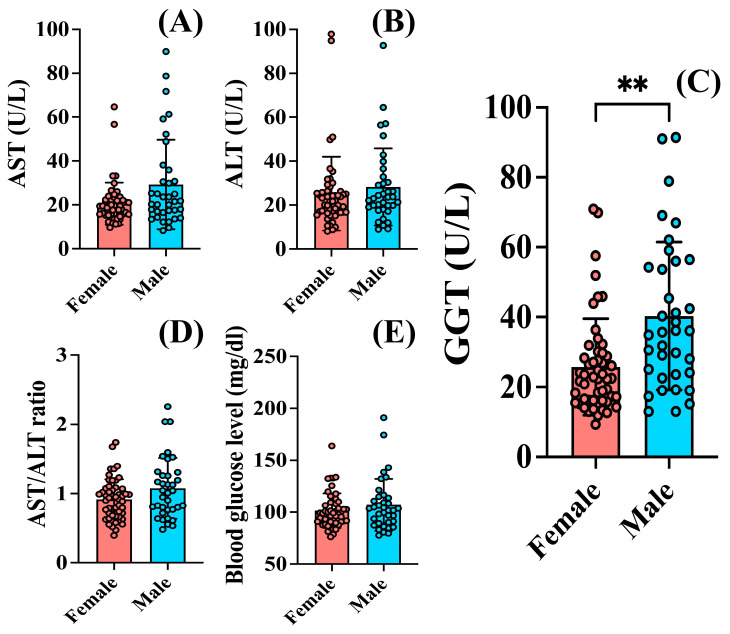
Gender differences in liver enzymes and blood glucose levels in patients with anxiety. There were no differences in (**A**) AST levels in male (29.32 ± 20.39 U/L), compared to female patients (20.55 ± 9.6 U/L) (*p* = 0.0678). Regarding (**B**) ALT, men had similar values (28.29 ± 17.55 U/L) to women (25.16 ± 16.77 U/L) (*p* > 0.05). Men showed increased (**C**) GGT levels (40.29 ± 21.17 U/L) compared to women (25.76 ± 13.81 U/L) (*p* = 0.0012). No differences in (**D**) AST/ALT ratio or (**E**) Blood glucose levels were observed. The graph shows mean values ± SD, ** *p* < 0.01.

Although for all analyzed parameters men had a tendency of higher average values compared to women, no differences were noticed in AST (Figure 3A) and ALT (Figure 3B) levels (*p* > 0.05). The same was observed for the AST/ALT ratio, with no differences between men (1.07 ± 0.43) and women (0.91 ± 0.29) (*p* > 0.05) (Figure 3D). Male patients showed similar blood glucose levels (107.1 ± 24.92 mg/dL) compared to females (101.5 ± 16.68 mg/dL) (*p* > 0.05) (Figure 3E).

Examining the impact of gender on GGT levels, we observed that men had higher values (40.29 ± 21.17 U/L) compared to women (25.76 ± 13.81 U/L) (*p* = 0.0012) (Figure 3C).

### 3.3. Gender Differences in GGT Levels Were Observed in Patients over 40 Years of Age

**Figure 4:**
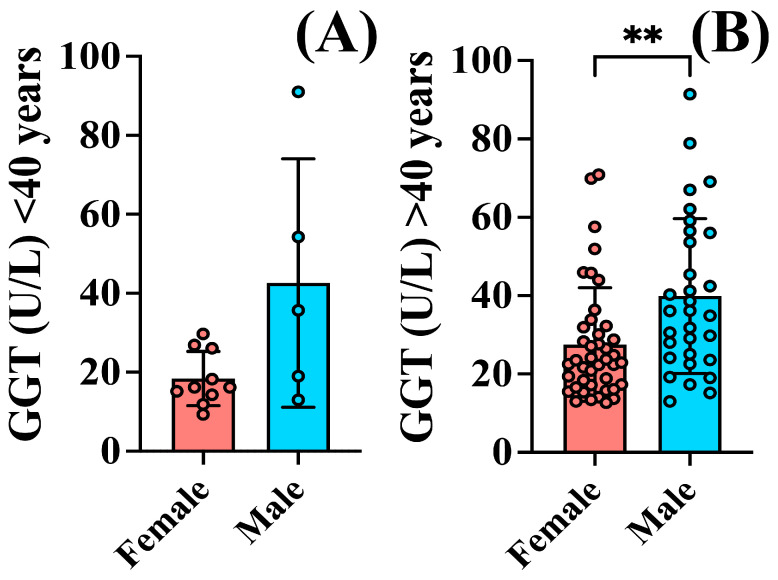
Gender differences in GGT levels in patients with anxiety disorders according to age. No difference in (**A**) GGT values between women (18.41 ± 6.84 U/L) and men (42.60 ± 31.46 U/L) under 40 years old (*p* = 0.1302). In (**B**) patients over 40 years of age, men showed higher GGT levels (39.91 ± 19.74 U/L) compared to women (27.50 ± 14.51 U/L) (*p* = 0.0084). The graph shows mean values ± SD, ** *p* < 0.01.

Out of the total patient cohort in this study, 5 men and 10 women were identified as being under 40 years old. After applying the nonparametric Kolmogorov–Smirnov test, while men tended to exhibit higher values (42.60 ± 31.46 U/L), no significant differences in GGT levels compared to women (18.41 ± 6.84 U/L) were observed upon analyzing the data (*p* = 0.1302) (Figure 4A).

In contrast, among patients aged over 40 years, men demonstrated elevated GGT values (39.91 ± 19.74 U/L), compared to female patients (27.50 ± 14.51 U/L) (*p* = 0.0084) (Figure 4B).

## 4. Discussion

### 4.1. Gender Differences in Anxiety Manifestation

The clinical manifestation of anxiety disorders in individuals of all ages is influenced by a combination of psychosocial and socio-environmental variables [61,62]. Research has shown that anxiety disorders have a greater impact on women [63], exhibiting a higher reactivity to anxiety [64]. The factors contributing to anxiety among women vary from concerns related to physical appearance and attractiveness in young women [65] to challenges related to physical disability and a perceived absence of emotional support among older ones [66]. During the luteal phase, more than 80% of women in their reproductive years have at least one physical, emotional, or anxiety symptom [67]. Furthermore, women transitioning into menopause often face emotional instability and are at a higher risk of developing anxiety disorders [68]. Conversely, men are more likely to exhibit externalizing behaviors when experiencing anxiety, such as irritability, anger, aggression, and alcohol abuse [36,69]. Certain cultural norms and expectations also play a role in men’s experiences of anxiety. Expressing vulnerability or seeking help could be seen as a sign of weakness [69], and men may be less likely to acknowledge or address their anxiety symptoms. Additionally, factors such as work-related stress, financial pressures [70,71], and relationship difficulties [72,73] can exacerbate anxiety in men. One of the limitations of this research is that we did not specifically address individual sex-related factors. However, with a gender ratio of approximately one man to two women affected by anxiety disorders, our findings support previous studies indicating that women are twice as likely as men to experience anxiety [63].

### 4.2. Impact of Alcohol Consumption on Liver Function

The patients included in this study showed anxiety symptoms severe enough to require hospitalization and displayed a significant functional impairment according to the GAF scale applied. Out of the total cohort of 88 patients, 15 were under the age of 40, aligning with previous findings indicating a lower probability for hospitalization among younger individuals compared to older age groups [74]. Alcohol consumption among patients with psychiatric disorders impacts their subsequent risk of hospitalization, with some gender differences already described [75]. While men suffering from anxiety tend to consume more alcoholic beverages than women [26,76], additional sex differences in alcohol metabolism and sensitivity to alcohol’s effects also contribute to variations in drinking patterns between men and women. Women generally have lower levels of alcohol dehydrogenase, the enzyme responsible for metabolizing alcohol [77], leading to higher blood alcohol concentrations and increased susceptibility to alcohol-related organ damage at lower levels of consumption compared to men [78]. Although patients with alcoholism were excluded from this study, the increase in the levels of liver enzymes in some of the patients suggests that they may underestimate their alcohol consumption. While men tended to exhibit higher average values for all analyzed parameters, elevated GGT levels, recognized as a biomarker associated with alcohol consumption [58], were significantly higher in men compared to women, regardless of treatment (Figure 3C). Upon analyzing the impact of age on GGT levels within our patient group, we noted gender discrepancies among individuals aged 40 years and above but no statistically significant variations were detected between genders among patients below the age of 40. This may be due to the limited number of patients included in the young analyzed group, or the decreasing discrepancy in alcohol consumption between genders, especially among younger individuals [37,38]. By adopting a gender-sensitive approach to alcohol prevention and treatment, clinicians can better support individuals in making healthier choices. Psychotherapeutic approaches, including cognitive-behavioral therapy [79], and exposure therapy [80], have demonstrated efficacy in reducing anxiety symptoms and improving coping mechanisms. Integrating these modalities with pharmacological treatments [81] may improve treatment response and long-term recovery.

### 4.3. Implications of Antidepressant Treatment

With antidepressant treatment having the potential to induce DILI [82], careful monitoring of both liver function and glucose metabolism during AD treatment is required [82,83]. Certain classes of antidepressants, such as TCAs and monoamine oxidase inhibitors (MAOIs), have been associated with a higher risk of hepatotoxicity compared to SSRIs or SNRIs [18]. However, cases of DILI have also been reported with the use of SSRIs such as sertraline [84], SNRIs including venlafaxine [85] and duloxetine [86], and in patients taking SARI-trazodone [87]. Conversely, SSRIs like citalopram and fluvoxamine are associated with a lower risk of DILI [18]. The combination of alcohol and antidepressants can potentiate central nervous system depression, leading to increased sedation, impaired cognitive function, and heightened risk of accidents or injuries [88]. Furthermore, alcohol can interfere with the metabolism of antidepressants, altering their pharmacokinetics and potentially reducing their efficacy or increasing the risk of adverse effects [89]. SSRIs and SNRIs, although generally considered safer in combination with alcohol compared to MAOIs and TCAs [90], still pose certain risks, both SSRIs [91,92] and SNRIs [93] potentially leading to serious health complications.

Among our patients, no differences in liver enzymes and blood glucose levels were observed between the various classes of ADs studied. Although no significance was reached, atypical TCA-treated patients showed a tendency for increased GGT values, especially compared to SSRIs (Figure 2C). This may suggest that the clinician had evidence to assume alcohol consumption, particularly given tianeptine’s efficacy in reducing alcohol intake [94,95]. This becomes more important as patients associating various pathologies could have an increased risk of liver injury. This is why we chose not to include them in the present study. With the elevation of ALT levels beyond three times the upper normal limit serving as a clinically significant indicator for DILI, this increase is often described in multidrug therapy and polypharmacy [82,83]. In our population, the observed liver changes can not be attributed to DILI, as all patients included in the present study received monotherapy medication prior to biological sampling. Furthermore, the average values of the measured biological parameters were within the accepted normal range. However, some patients did show elevated ALT values, cases in which DILI cannot be completely excluded, although they did not exhibit other clinical-specific symptoms such as nausea, abdominal pain, pruritus, jaundice, and ascites [96] during the study interval. Future research with larger, prospective studies is needed to explore the long-term effects of antidepressant treatment or combined drug therapy on liver function in patients with anxiety disorders.

### 4.4. Glycemic Control

Although a conclusive link between the use of antidepressants and an increased likelihood of glycemic control irregularities has not been demonstrated [97], there are reports linking the use of antidepressants to impaired glycemic control [98]. SSRIs have the potential to induce hypoglycemia by disrupting the central mechanisms responsible for triggering hormonal responses that counteract low blood sugar levels [99]. While the use of antidepressants and glycemia is still debated, there is a clear association between treatment and alcohol consumption, leading to either hypo- or hyperglycemia [28,29]. No significant disparities were seen in blood glucose levels across the various types of ADs within our patient population. This may be due to the limited number of patients included in the study. However, a subgroup of treated patients in all classes of ADs had blood glucose levels that exceeded the normal limit. Future investigations are needed for a clearer perspective regarding glycemic control and assessment of liver status in patients undergoing antidepressant treatment for anxiety disorders.

### 4.5. Limitations and Future Directions

The smaller sample size in some age groups, especially younger individuals, may have influenced the statistical power of our analyses and limited our ability to detect significant differences in liver enzymes and blood glucose levels between genders among patients below the age of 40. Additionally, the exclusion of patients with alcoholism or other comorbidities may have affected the generalizability of our findings. However, our findings offer valuable insights into the coping mechanisms of patients with moderate alcohol consumption and severe anxiety disorders, in monotherapy with antidepressants. Although our findings did not indicate a significant difference in liver enzymes across different classes of antidepressants, increased GGT values were observed in men over 40 years old compared to women, regardless of treatment.

Larger prospective studies are warranted to elucidate the long-term effects of antidepressant treatment on liver function and glycemic control in patients with anxiety disorders. These investigations should track patients over an extended duration to gauge the cumulative impacts of antidepressant use. Future research should explore the differential impact of antidepressant treatment on liver function and glycemic control across various age brackets and genders. Understanding the influence of these demographic factors on the response to antidepressant treatment and the potential for adverse effects can facilitate the tailoring of treatment strategies to specific patient cohorts. The present study aims to examine the link between severe anxiety disorders and liver dysfunction. Although we are confident in our reporting, it is important to highlight that this study’s main purpose is investigational and analytical, and it is not intended to offer treatment guidance for clinicians or dictate clinical decision-making processes. Rather, the findings should be seen as the basis for further research on the topic, due to the increased pressure from the ever-increasing prevalence of chronic disease coexistence. Of note is that all analysis treatments are approved and are being used in anxiety patients, and while our results show that male patients underestimate their alcohol consumption, this might be true only for the studied population and may not apply to other populations with different social norms.

A multidisciplinary approach involving psychiatrists, hepatologists, and endocrinologists is indispensable in managing patients with anxiety disorders, alcohol consumption, and related comorbidities due to the intricate interconnections between these conditions at physiological and biochemical levels. Psychiatrists are responsible for the accurate diagnosis and management of anxiety disorders. Hepatologists play a critical role in evaluating and managing liver damage caused by alcohol consumption. Moreover, liver dysfunction can affect the metabolism and clearance of hormones, such as insulin, thyroid hormones, and sex hormones [100], necessitating specialized endocrine evaluation and management. This collaborative approach ensures optimal management of each aspect of the patient’s health, leading to improved clinical outcomes and quality of life. Additionally, regular interdisciplinary communication facilitates early detection of potential complications and timely interventions, thereby reducing morbidity and mortality associated with these complex and intertwined conditions.

## 5. Conclusions

Among patients with anxiety disorders severe enough to require hospitalization, increased GGT values were observed in men compared to women, indicating that alcohol consumption, as a coping mechanism, is gender specific. However, this difference between genders was not observed among patients under the age of 40. While gender differences observed in alcohol consumption can have an impact on liver function, glycemic control does not appear to be as rigorously necessary.

Our study underscores the importance of early detection of alcohol consumption in patients with anxiety disorders, as this can impact their liver function and overall health. Clinicians should be vigilant in screening for alcohol consumption and monitoring liver function in patients presenting with anxiety symptoms, especially considering the gender-specific differences in alcohol consumption and its impact on liver enzymes.

## Figures and Tables

**Table 1:** Descriptive statistics of patients based on their treatment.

AD	Number of Patients	Mean Age (years)	Gender Ratio (M:F)	AST Blood Levels (1–40 U/L)	ALT Blood Levels (1–41 U/L)	AST/ALT Ratio (<1.1)	GGT Blood Levels (1–38 U/L)	Blood Glucose Levels (70–110 mg/dL)
**Total**	88	50.00 ± 11.15	36:52	24.14 ± 15.50	26.44 ± 17.06	0.98 ± 0.36	31.70 ± 18.54	103.8 ± 20.51
**SSRI**	29	48.62 ± 13.63	9:20	21.32 ± 9.51	24.00 ± 10.78	0.97 ± 0.37	27.76 ± 15.43	109.2 ± 22.81
**SNRI**	26	49.50 ± 9.27	13:13	25.08 ± 16.64	28.05 ± 21.44	0.95 ± 0.33	31.25 ± 18.68	98.33 ± 14.60
**TCA**	17	50.47 ± 9.05	9:8	28.89 ± 22.82	26.74 ± 13.19	1.08 ± 0.46	38.77 ± 22.23	98.98 ± 25.39
**SARI**	16	52.81 ± 11.40	5:11	22.66 ± 12.76	27.93 ± 22.39	0.92 ± 0.28	32.06 ± 18.78	107.8 ± 16.55

**Table 2:** Descriptive statistics of the studied population and normal distribution testing.

		AST Blood Levels (U/L)	ALT Blood Levels (U/L)	AST/ALT Ratio	GGT Blood Levels (U/L)	Blood Glucose Levels (mg/dL)
**Descriptive statistics of the studied population**	**Mean**	24.14	26.44	0.98	31.7	103.8
	**Std. Deviation**	15.5	17.1	0.4	18.5	20.5
	**Minimum**	8.3	8.1	0.4	9.3	76.3
	**Maximum**	89.9	97.9	2.3	91.4	191.1
	**Range**	81.6	89.8	1.9	82.1	114.8
	**Skewness**	2.4	2.5	1.1	1.4	1.8
	**Kurtosis**	5.7	7.5	1.6	1.5	4.5
**Test for normal distribution D’Agostino and Pearson test**	**K2**	57.7	64.6	20.3	24.3	43.3
	***p* value**	<0.0001	<0.0001	<0.0001	<0.0001	<0.0001
	**Passed normality test (alpha = 0.05)?**	No	No	No	No	No

## Data Availability

The data presented in this study are available upon request from the corresponding authors.
